# Fundoplication for Pediatric Gastroesophageal Reflux Disease: Indications, Techniques, and Outcomes

**DOI:** 10.7759/cureus.72930

**Published:** 2024-11-03

**Authors:** Maimona A Al-Refaie, Mohammed M Alsurmi, Yasser A Obadiel, Haitham M Jowah, Khaled M Alsharafy

**Affiliations:** 1 Department of Pediatric Surgery, Al-Thawra Modern General Hospital, Sana'a, YEM; 2 Department of Pediatric and Neonatal Surgery, Al-Thawra Modern General Hospital, Sana'a, YEM; 3 Department of Surgery, Faculty of Medicine and Health Sciences, Sana'a University, Sana'a, YEM; 4 Department of Surgery, Faculty of Medicine and Health Sciences, Sana’a University, Sana'a, YEM

**Keywords:** esophageal stricture management, fundoplication success rates, gerd treatment, nissen fundoplication, pediatric gastroesophageal reflux disease, pediatric surgery outcomes, postoperative complications, thal fundoplication

## Abstract

Background

This study aimed to evaluate the indications, techniques, and outcomes of fundoplication in pediatric patients with gastroesophageal reflux disease (GERD) at a tertiary hospital in Yemen.

Methods

A prospective cohort study was conducted at Al-Thawra Modern General Hospital, Sana’a, Yemen, between January 2015 and January 2022. The study included 45 pediatric patients under 18 years of age who underwent fundoplication for GERD. Data on demographic and clinical characteristics, surgical type, postoperative complications, and follow-up outcomes were collected and analyzed.

Results

The median age of the 45 pediatric patients was two years (range: two months to 10 years), with 25 males (56%) and 20 females (44%). The primary indications for fundoplication included GERD with hiatal hernia in 20 patients (44.4%), persistent symptoms despite medical management in 9 patients (20%), recurrent chest infections associated with neurological disorders in 7 patients (15.6%), esophageal stricture in 8 patients (17.8%), and both hiatal hernia and esophageal stricture in 1 patient (2.2%). Nissen fundoplication was performed in 36 patients (80%), and Thal fundoplication was performed in 9 patients (20%). Complete symptom resolution was achieved in 31 patients (68.89%). Dysphagia due to postoperative esophageal stricture was the most common complication, affecting 13 patients (29%). A structured dilatation protocol resulted in improvement after one session in four patients (30.8%), regular sessions in six patients (46.2%), and irregular sessions in three patients (23.1%). The mortality rate was two patients (5.56%), both of whom had cerebral palsy and died due to recurrent chest infections unrelated to GERD recurrence or esophageal stricture.

Conclusions

Fundoplication is a safe and effective surgical treatment for pediatric GERD, with a high success rate and manageable complications. A structured postoperative dilatation protocol is essential for managing esophageal strictures and improving outcomes. Early diagnosis and intervention, alongside adherence to postoperative protocols, are crucial for optimal results. Further research with larger sample sizes and long-term follow-up is recommended to confirm these findings and improve clinical practice.

## Introduction

Gastroesophageal reflux disease (GERD) is a common condition among pediatric patients and is characterized by the backward flow of stomach acid into the esophagus, causing discomfort and potential damage [1-3]. In severe cases, GERD can lead to complications such as esophageal stricture, Barrett’s esophagus, and aspiration pneumonia. Medical management is typically the first-line treatment, but a subset of patients fail to respond adequately to pharmacological interventions. For these patients, surgical options, such as fundoplication, become necessary [4-6].

Fundoplication has been widely adopted as a surgical solution for pediatric GERD patients who are unresponsive to medical management [7]. The procedure involves wrapping the upper part of the stomach around the lower esophagus to reinforce the lower esophageal sphincter and prevent acid reflux. There are several variations of fundoplication, including Nissen (360-degree wrap), Thal (partial anterior wrap), and Toupet (partial posterior wrap) [4,5]. The choice of technique depends on the patient's specific condition and the surgeon's preference.

While fundoplication is generally considered safe and effective, the outcomes can vary based on several factors, including the presence of preoperative esophageal stricture, hiatal hernia, and the patient's overall health condition [8-10]. Previous studies have reported success rates ranging from 70% to 80%, with variations in postoperative complications such as dysphagia and wrap migration [11-13]. However, comprehensive data on the indications, techniques, and outcomes of fundoplication in pediatric patients from resource-limited settings are scarce.

This study aimed to fill this gap by evaluating the indications for fundoplication, surgical techniques, and postoperative outcomes in pediatric patients with gastroesophageal reflux disease treated at Al-Thawra Modern General Hospital in Sana’a, Yemen. By analyzing a cohort of patients over a seven-year period, we provide valuable insights into the effectiveness and safety of fundoplication in this specific context.

This article was previously posted as a preprint on the Research Square preprint server on July 30, 2024.

## Materials and methods

Study design and setting

This study employed a prospective cohort design conducted at Al-Thawra Modern General Hospital, a tertiary care center in Sana’a, Yemen. The study spanned from January 2015 to January 2022, focusing on evaluating the indications, surgical techniques, and outcomes of fundoplication in pediatric patients diagnosed with gastroesophageal reflux disease (GERD). The Institutional Review Board of Al-Thawra Modern General Hospital granted ethical approval (Approval No. IRB/THW/2014/0123). We obtained informed consent from the parents or guardians of all participants.

Participants

The study included pediatric patients younger than 18 years who underwent fundoplication for GERD. To be eligible for the study, people had to meet three conditions: (1) they had to have been diagnosed with GERD through a clinical evaluation and diagnostic investigations; (2) their medical treatment had failed or they had GERD-related complications like an esophageal stricture or a hiatal hernia; and (3) they had to have significant neurological impairment, such as severe cerebral palsy, or a history of recurrent respiratory infections. We excluded patients with incomplete medical records or those who lost follow-up during the study period.

Preoperative evaluation

We conducted a comprehensive preoperative assessment for all patients, which included a detailed clinical history, physical examination, and diagnostic imaging. We systematically performed upper gastrointestinal endoscopy to evaluate the esophageal mucosa, confirm the diagnosis of GERD, and rule out other potential etiologies of symptoms. We used barium swallow studies to assess structural abnormalities such as hiatal hernia and esophageal stricture. Pediatric surgeons, neurologists, and pulmonologists performed a multidisciplinary evaluation for neurologically impaired patients to optimize preoperative care and address factors such as feeding difficulties and respiratory risk.

Surgical procedures

Experienced pediatric surgeons performed the fundoplication under general anesthesia. We selected the surgical technique - Nissen or Thal fundoplication - based on clinical presentation, anatomical factors, and patient-specific considerations.

Nissen Fundoplication

This procedure involved a complete 360-degree wrap of the gastric fundus around the lower esophagus to strengthen the lower esophageal sphincter [12,14]. Patients with significant anatomical abnormalities, such as hiatal hernia or severe GERD symptoms necessitating a robust anti-reflux barrier, were the ones who chose it. We performed intraoperative esophageal dilatation alongside Nissen fundoplication in cases where a preoperative esophageal stricture was present.

Thal Fundoplication

Patients with smaller gastric sizes, milder GERD, or the risk of postoperative dysphagia were candidates for a partial anterior 180-degree wrap of the stomach. Patients with minimal anatomical disruption or when a complete wrap posed a technical challenge favored this technique [8].

The decision-making process for selecting the surgical approach was based on individualized patient assessments, including the severity of symptoms, anatomical findings, and underlying comorbidities.

Postoperative management and follow-up

Postoperative care followed standardized protocols, with patients monitored for immediate postoperative complications, including dysphagia, respiratory distress, and pain. Routine postoperative evaluations occurred at two weeks, two months, six months, and one year following surgery, including clinical assessments and diagnostic imaging. We performed barium swallow studies two months postoperatively and conducted upper gastrointestinal endoscopy at two to three months, or earlier if clinically indicated, to identify complications such as esophageal stricture.

We implemented a structured dilatation protocol for patients who developed postoperative esophageal strictures. We followed the initial intraoperative dilatation with regular sessions starting one month after surgery, performing weekly dilations until we achieved a stable dilator size. After confirming stability, we performed dilations every month for four months and then every two months for the first postoperative year. We monitored progress through endoscopic and radiological evaluations.

Data collection

We collected data using standardized forms and comprehensive reviews of medical records. Information gathered included demographic characteristics, clinical presentation, surgical details (type of fundoplication), intraoperative findings, postoperative complications, and follow-up outcomes. Primary outcomes were defined as the success rate of fundoplication, measured by complete symptom relief during the one-year follow-up. Secondary outcomes included the frequency of postoperative complications, the need for esophageal dilatation, and mortality.

Statistical analysis

We analyzed all data using IBM SPSS Statistics, version 26.0 (IBM Corp., Armonk, NY, US). We employed descriptive statistics to summarize demographic data, clinical characteristics, and outcomes. Depending on the data distribution, we reported continuous variables as means with standard deviations or medians with interquartile ranges. We presented category variables as frequencies and percentages. Associations between categorical variables were assessed using the chi-square test, with a significance level set at p < 0.05. The main things that were compared between the Nissen and Thal fundoplication outcomes were complications after surgery, like dysphagia, the need for dilation after surgery, and the rates at which symptoms went away. We acknowledged the statistical limitations resulting from sample size differences between the two surgical groups.

## Results

A total of 45 pediatric patients underwent fundoplication for GERD at Al-Thawra Modern General Hospital between January 2015 and January 2022. The median age of the patients was two years (range: two months to 10 years), with 25 males (56%) and 20 females (44%). The primary symptoms were vomiting in all patients (45 patients, 100%) and regurgitation in 21 patients (47%) (Table 1).

**Table 1:** Demographic and clinical characteristics of the study population Data are represented as the number of patients (N) and percentages (%). GERD, gastroesophageal reflux disease; N, number of patients; %, percentage

Variable	N	%
Gender		
Female	20	44
Male	25	56
Age		
< 1 year	8	18
1-3 years	16	36
3-6 years	1	2
6-12 years	20	44
Clinical Symptoms		
Vomiting	45	100
Regurgitation	21	47
Recurrent Chest Infection	7	16
Epigastric Pain	2	4.5
Comorbidity		
Neurological Disorder (Cerebral Palsy)	7	15.6
Preoperative Esophageal Stricture	9	20
Preoperative Investigation		
Upper Endoscopy	35	78
Barium Swallow	45	100
Preoperative Diagnosis		
GERD With Hiatal Hernia	20	44
GERD Without Esophageal Stricture	16	36
GERD With Esophageal Stricture	8	18
GERD With Hiatal Hernia + Esophageal Stricture	1	2

The primary indications for fundoplication were GERD with hiatal hernia in 20 patients (44.4%), persistent symptoms despite medical management in nine patients (20%), recurrent chest infections associated with neurological disorders in seven patients (15.6%), esophageal stricture in eight patients (17.8%), and both hiatal hernia and esophageal stricture in one patient (2.2%) (Table 2).

**Table 2:** Indications for fundoplication among the study population (n=45) Data are represented as the number of patients (N) and percentages (%). GERD, gastroesophageal reflux disease; N, number of patients; %, percentage

Indication	N	%
GERD with hiatal hernia	20	44.4
GERD with persistent symptoms despite medical management	9	20
GERD in patients with cerebral palsy and recurrent chest infections	7	15.6
GERD with esophageal stricture	8	17.8
GERD with hiatal hernia + esophageal stricture	1	2.2

Nissen fundoplication was performed in 36 patients (80%), whereas Thal fundoplication was performed in nine patients (20%). Fundoplication with hiatal hernia repair was conducted in 20 patients (44.4%), fundoplication alone in 16 patients (35.6%), and fundoplication with postoperative dilatation in nine patients (20%) (Table 3).

**Table 3:** Distribution of surgical procedures according to fundoplication type Data are represented as the number of patients (N) and percentages (%).

Surgical Procedures	Nissen Fundoplication (n = 36, 80%)	Thal Fundoplication (n = 9, 20%)	Total (n = 45)
Fundoplication with hiatal hernia repair	14 (39%)	6 (67%)	20 (44%)
Fundoplication alone	13 (36%)	3 (33%)	16 (36%)
Fundoplication with postoperative dilatation	8 (22%)	0	8 (18%)
Fundoplication with hiatal hernia repair + postoperative dilatation	1 (3%)	0	1 (2%)

Postoperative complications were observed in 20 patients (44%). The most common complication was dysphagia due to postoperative esophageal stricture, affecting 13 patients (28.9%). Other complications included epigastric pain in two patients (4.4%), chest infections in four patients (8.9%), and a sense of fullness in one patient (2.2%) (Table 4).

**Table 4:** Postoperative complications among the study population (n=45) Data are represented as the number of patients (N) and percentages (%).

Postoperative Complications	N	%
Epigastric pain	2	4
Fullness	1	2
Chest infection	4	9
Dysphagia (esophageal stricture)	13	29
Total	20	44

During the one-year follow-up, 31 patients (68.9%) experienced complete symptom resolution. Among those who required postoperative dilatation, four patients (30.8%) improved after a single session, six patients (46.2%) improved after regular sessions, and three patients (23.1%) did not improve after irregular sessions. The overall mortality rate was 5.6% for two patients, both with cerebral palsy who died due to recurrent chest infections unrelated to GERD recurrence or esophageal stricture (Table 5).

**Table 5:** Postoperative Outcomes of the Study Population (n = 45) Data are represented as the number of patients (N) and percentages (%).

Postoperative Outcomes	N	%
Complete symptom improvement	31	69
Postoperative stricture dilation	13	29
Postoperative dilatation outcomes (n=13)		
Improved after one session	4	30.8
Improved after regular sessions	6	46.2
Not improved after irregular sessions	3	23.1
Redo operation	1	2
Mortality	2	4

Of the 45 patients who underwent fundoplication, 36 received Nissen and 9 received Thal procedures. Dysphagia was reported in 13 patients (36.11%) in the Nissen group and none in the Thal group (p = 0.034). There were no significant differences between the groups for epigastric pain (1 patient, 2.78% in Nissen vs. 1 patient, 11.11% in Thal), fullness (1 patient, 2.78% in Nissen vs. 0 in Thal), or chest infections (4 patients, 11.11% in Nissen vs. 0 in Thal) (p > 0.05 for all). Complete resolution of symptoms was achieved in 22 patients (61.11%) in the Nissen group and 9 patients (100%) in the Thal group, approaching significance (p = 0.060). The need for postoperative dilatation was significantly higher in the Nissen group (13 patients, 36.11%) compared to none in the Thal group (p = 0.034). No significant differences were observed for redo operations (1 patient, 2.78% in Nissen vs. 0 in Thal), improvement after dilatation (10 patients, 27.78% in Nissen vs. 0 in Thal), or mortality (2 patients, 5.56% in Nissen vs. 0 in Thal) (p > 0.05 for all) (Table 6).

**Table 6:** Comparison of postoperative outcomes between Nissen and Thal procedures in the study population Data are represented as the number of patients (N) and percentages (%). P-values were calculated using the chi-square test, and a p-value < 0.05 was considered statistically significant, indicated by an asterisk (*).

Variable	Nissen Fundoplication (n = 36)	Thal Fundoplication (n = 9)	Total (n = 45)	Chi-Square Value	P-value
Postoperative Complications					
Dysphagia	13 (36.11%)	0 (0%)	13 (29%)	4.49	0.034 *
Epigastric Pain	1 (2.78%)	1 (11.11%)	2 (4%)	0.225	0.635
Fullness	1 (2.78%)	0 (0%)	1 (2%)	0.026	0.873
Chest Infection	4 (11.11%)	0 (0%)	4 (9%)	1.62	0.203
Postoperative Outcomes					
Complete Resolution of Symptoms	22 (61.11%)	9 (100%)	31 (69%)	3.53	0.060
Redo Operation	1 (2.78%)	0 (0%)	1 (2%)	0.03	0.864
Need for Postoperative Dilatation	13 (36.11%)	0 (0%)	13 (29%)	4.49	0.034 *
Improvement After Dilation	10 (27.78%)	0 (0%)	10 (22%)	1.93	0.165
Mortality	2 (5.56%)	0 (0%)	2 (4%)	0.36	0.548

## Discussion

This study aimed to evaluate the indications, techniques, and outcomes of fundoplication in pediatric patients with GERD treated at a tertiary hospital in Yemen. The findings provide valuable insights into the effectiveness of fundoplication and highlight the importance of specific indications for achieving optimal outcomes.

The demographic characteristics of our study population, with a median age of two years and a nearly equal sex distribution (25 males, 56%, and 20 females, 44%), are consistent with other studies on pediatric patients undergoing fundoplication for GERD. This demographic consistency strengthens the reliability of our findings and their applicability to similar patient populations [15].

The primary indications for fundoplication in our study included GERD with hiatal hernia in 20 patients (44.4%), persistent symptoms despite medical management in 9 patients (20%), recurrent chest infections associated with neurological disorders in 7 patients (15.6%), esophageal stricture in 8 patients (17.8%), and both hiatal hernia and esophageal stricture in 1 patient (2.2%). These indications align with those reported in the literature, where fundoplication is commonly indicated for severe GERD symptoms unresponsive to medical therapy, anatomical abnormalities, and GERD-related respiratory complications [12,16]. This underscores the necessity of surgical intervention in patients with these specific conditions to prevent further complications and improve quality of life.

In terms of outcomes, our study revealed that 31 patients (68.9%) experienced complete symptom resolution after surgery. This success rate is comparable to those reported in other recent studies, where symptom resolution rates ranged from 60% to 90% among pediatric patients undergoing fundoplication [15,17]. Postoperative complications were observed in 15 patients (33.3%), with dysphagia due to postoperative esophageal stricture being the most common complication, affecting 13 patients (28.9%). Among these patients, nine patients (20%) had preoperative esophageal strictures, and four patients (8.9%) developed dysphagia after Nissen fundoplication with hiatal hernia repair. These findings emphasize the need for careful patient selection and postoperative management [18,19].

Our structured dilatation protocol for managing postoperative esophageal strictures played a crucial role in the success of our fundoplication procedures. Specifically, four patients (30.8%) improved after a single session, six patients (46.2%) improved after regular sessions, and three patients (23.1%) did not improve after irregular sessions. These findings are consistent with several relevant studies on postfundoplication dilatation. For example, Malhi-Chowla et al. (2002) reported that 91% of patients who underwent esophageal dilatation after fundoplication experienced symptomatic relief, highlighting the efficacy of regular dilatation sessions [20]. Similarly, Spivak et al. (1998) emphasized the importance of follow-up and regular intervention, noting that postoperative re-dilatation was necessary to achieve satisfactory outcomes in managing strictures [21]. Koivusalo and Pakarinen (2018) analyzed pediatric fundoplication and reported that postoperative esophageal strictures were effectively managed with a structured follow-up protocol, aligning with our findings that regular sessions yield better outcomes than irregular management [22]. In contrast, El-Serag and Sonnenberg (1999) noted that postoperative dysphagia and strictures are common complications requiring consistent management to prevent recurrence and improve patient outcomes [23]. Csendes et al. (2019) conducted a long-term follow-up study on patients who underwent laparoscopic Nissen fundoplication and reported that timely and regular dilatation procedures were effective in managing esophageal strictures [24]. These studies reinforce the importance of structured postoperative care plans to mitigate the risks of esophageal strictures and ensure optimal patient outcomes.

In our study, the comparison between Nissen fundoplication and Thal fundoplication demonstrated better outcomes with Thal fundoplication, including fewer cases of dysphagia (P = 0.034) and a higher rate of complete symptom resolution (P = 0.06). Specifically, dysphagia was observed in 13 patients (36.11%) in the Nissen group, whereas it was not reported in the Thal group. Additionally, 22 patients (61.11%) in the Nissen group achieved complete symptom resolution, whereas nine patients (100%) in the Thal group achieved symptom resolution. These results may be partly explained by our selection criteria, where Nissen fundoplication was performed in patients with preoperative esophageal strictures, whereas Thal fundoplication was used in patients with smaller stomachs. This clinical decision-making may have introduced selection bias, contributing to the observed differences in outcomes. However, these differences did not reach statistical significance.

Comparative studies have shown that while Nissen fundoplication is effective, it is associated with a higher incidence of postoperative complications, such as dysphagia and bloating, than other techniques are [25]. These findings suggest that alternative fundoplication techniques may reduce specific complications, although overall success rates remain comparable [26,27].

The observed mortality rate was 5.56% in our study, with two deaths occurring six months after Nissen fundoplication in patients with cerebral palsy, underscoring the significant risks associated with this surgical intervention in neurologically impaired individuals. These fatalities were due to recurrent chest infections related to their underlying neurological conditions rather than GERD recurrence or esophageal stricture. This finding aligns with previous studies documenting the heightened vulnerability of neurologically impaired patients to respiratory complications following fundoplication, emphasizing the need for comprehensive preoperative assessment, vigilant postoperative monitoring, and a multidisciplinary approach [28,29].

These findings have important clinical implications. Fundoplication should be considered a viable option for pediatric patients with GERD who are unresponsive to medical management. Early diagnosis and intervention, combined with regular postoperative follow-up and dilatation protocols, are crucial for improving outcomes, particularly in patients with preoperative esophageal strictures. Surgeons should consider individual patient anatomy and clinical conditions when selecting the type of fundoplication. Additionally, addressing socioeconomic barriers to adherence could further enhance patient outcomes.

Future research should focus on long-term outcomes of fundoplication in pediatric patients, including quality-of-life assessments and predictors of successful outcomes. Comparative studies between different fundoplication techniques and the role of minimally invasive approaches in pediatric populations could provide valuable insights. Multicenter studies could enhance the generalizability of findings and provide a broader perspective on the effectiveness of fundoplication in diverse populations.

Strengths and limitations

A strength of this study is the comprehensive inclusion of various indications for fundoplication, providing a detailed overview of its efficacy across different patient subsets. However, limitations include the lack of 24-hour esophageal pH monitoring and the reliance on barium studies and endoscopies, which may affect the precision of our findings. Socioeconomic factors also influence adherence to postoperative protocols, particularly regular dilatation, which may impact outcomes. Additionally, the observational nature and single-center design of this study may limit the generalizability of the results.

## Conclusions

This study confirms that fundoplication is an effective surgical treatment for pediatric patients with GERD who do not respond to medical management. Thal fundoplication demonstrated better outcomes than Nissen did, with fewer cases of dysphagia and higher rates of symptom resolution, although the differences were not statistically significant. A structured dilatation protocol is crucial for managing postoperative esophageal strictures, highlighting the importance of regular and timely interventions. The observed mortality in patients with cerebral palsy underscores the need for comprehensive preoperative assessment and vigilant postoperative monitoring. Further research should explore long-term outcomes, comparative techniques, and strategies to improve adherence to postoperative protocols to enhance patient care and outcomes.
